# Lessons learned from implementing a digital rehabilitation care planning platform to improve care access for patients with work disability: qualitative process evaluation of the RehaPro-SERVE study

**DOI:** 10.1186/s12913-024-11778-3

**Published:** 2024-10-29

**Authors:** Kristina Buch, Viktoria Hamme, Annette Becker, Ulf Seifart, Catharina Maulbecker-Armstrong, Karin Moser, Pellumbesha Seferi, Antonia Keller, Veronika van der Wardt

**Affiliations:** 1 Department of Primary Care, Philipps-University of Marburg, Karl- von-Frisch-Straße 4, Marburg, 35032 Germany; 2 Sonnenblick Medical Rehabilitation Centre, German Pension Insurance, Amöneburger Straße 1-6, Marburg, 35043 Germany; 3 Faculty of Health Sciences, University of Applied Sciences Central Hesse, Wiesenstraße 14, Giessen, 35390 Germany; 4 German Pension Insurance of Hesse, Städelstraße 28, Frankfurt am Main, 60596 Germany

**Keywords:** Rehabilitation, Primary care, Case management, Case conferences, Interdisciplinary care planning, Complex intervention, Digital platform, Process evaluation

## Abstract

**Background:**

Inpatient rehabilitation therapies can be applied for in Germany by patients of working age to support their return to work. However, there are some problems that impede an easy and uncomplicated application process. An interdisciplinary case management approach for rehabilitation care planning was developed to facilitate the access to rehabilitation. Case conferences (CCs) were held with relevant stakeholders and took place on a digital communication platform. We conducted a qualitative process evaluation to understand the implementation of the intervention and to identify contextual factors as well as mechanisms for a successful implementation in the context of primary care.

**Methods:**

The process evaluation included interviews with primary care physicians (PCPs), patients and stakeholders involved in the intervention process. Reflexive thematic analysis was used to analyse the data. Emerging themes were structured according to the Donabedian framework of structure, process and outcomes.

**Results:**

A total of 18 interviews were conducted. Important results included the desire for more patient involvement and case management. Patients especially valued the opportunity to receive support from a social worker. Limitations of the platform related to usability and limited opportunities for stakeholder communication. Despite training for PCPs, several problems arose regarding the clarity of the intervention process. Patients were satisfied with their application process and the treatments offered, while PCPs reported an increase in workload.

**Conclusions:**

A digitalisation of the application procedure for rehabilitation and further treatment options is acceptable to patients and personal support of a social worker is particularly valued. However, patients should be included in the CC in terms of a shared decision-making process. The digital platform requires sufficient training and adjustments have to be made to enhance usability and to improve the efficiency of the process for PCPs. Overall, the exchange between the various stakeholders in the CC is considered particularly useful in more complex cases.

**Trial registration:**

DRKS German Clinical Trials Register, DRKS0 00242 07. Registered on 22 March 2021.

**Supplementary Information:**

The online version contains supplementary material available at 10.1186/s12913-024-11778-3.

## Background

Rehabilitation therapies are an effective means of helping patients with chronic conditions to return to work [1–3] and can be applied for in Germany by patients of working age from the German pension insurance, usually responsible for funding medical rehabilitation. In this case, applications are made in consultation with the patient’s primary care physician (PCP) [4, 5] who is assuming a gatekeeper position. Usually, the German pension insurance is responsible for the approval or rejection of the application, which is based on an examination of personal (e.g. medical necessity assessed by the attending practitioner, working ability significantly reduced or at risk) and insurance requirements (e.g. fulfilment of insurance and contribution periods).

While outpatient medical rehabilitation is the predominant form of rehabilitation in Europe, inpatient rehabilitation accounts for about 80% of all approved rehabilitation services in Germany [6]. Usually, patients are treated in inpatient rehabilitation clinics for a period of three weeks [7] and treatments can consist of various components such as medical treatment, exercise therapy (physiotherapy, sports therapy), work-related measures, health and patient education, psychological counselling, relaxation methods, occupational therapy, physical therapy, nutritional counselling as well as social, socio-legal and occupational counselling [8].

However, there are a number of problems in this process that impede the easy and uncomplicated access to rehabilitative therapies. Due to the requirements mentioned above, the application and approval process as well as rehabilitation therapies themselves only allow for a small degree of individualisation. Further, the application and approval process lacks multidisciplinary communication (e.g. between PCP practices and physicians in the German pension insurance). This leads to a loss of transparency perceived by PCPs in the decision-making process [4, 9, 10], e.g. with regard to the processing status of applications, the comprehensibility of the approval or rejection of applications and the criteria for such a decision [9]. Multidisciplinary approaches to care planning have been shown to improve functional outcomes for patients with complex needs [11] and strategies to improve the communication between service providers are associated with improved health outcomes and patient satisfaction [12]. Digital and platform-based approaches offer a way to facilitate the communication of various stakeholders [13–15]. The RehaPro-SERVE study and the respective complex intervention [16] were designed to facilitate rehabilitation care planning using an interdisciplinary and digitalised case management approach.

## The RehaPro-SERVE study and intervention

The RehaPro-SERVE study was a pragmatic, multicentre, randomised controlled two-arm parallel-group superiority trial. The overall objective was to assess the effectiveness of the intervention compared to treatment/ care planning as usual (control condition) in terms of a reduction in the number of days of sick leave and an increased work ability. For patients allocated to the intervention group interdisciplinary (rehabilitation) care planning took place in case conferences (CC), arranged on a digital communication platform. Due to insufficient recruitment numbers the RCT was terminated early. Before the start of the CC patients answered the Work Ability Index (WAI) [17], the Würzburger Screening, used to assess work-related problems which are relevant for the conduct of medical rehabilitation [18] and further medical anamnesis questionnaires to help the decision on an appropriate treatment. The CCs were then held with relevant stakeholders and took place on a digital communication platform with communication in writing to ensure fast but asynchronous communication without the need for stakeholders to meet in person. In the CCs, the attending PCP and a public health physician from the German pension insurance were involved as experts for their patients as well as their wishes or for rehabilitation care planning, as well as employees from employment agencies or job centers. Before conducting their first CC, PCPs received training regarding the purpose and procedures for the CC as well as the use of the platform by a member of the study team. Besides inpatient rehabilitation stakeholders could propose various treatment options (Additional file 1), which were then considered by the others until a consensus was reached. The CCs as well as agreed treatments were arranged by a case administrator, employed from the German pension insurance to facilitate stakeholder communication. A social worker, also employed by the German pension insurance, could be activated by clicking a button on the platform to support patients where necessary. The case administrator, the social worker and patients did not take part in the CC themselves. Table 1 provides an overview of the different stakeholders involved in the CC and the overall intervention process. The intervention aimed to facilitate rehabilitation care planning through 3 mechanisms: (1) the effective and interdisciplinary communication of the stakeholders (2) the tailoring of treatment offers and (3) the possibility for additional patient support by a social worker. In addition to an individualised selection of treatment options, the intervention also enabled the provision of services from employment agencies and job centers as well as the provision of treatments for which patients would not meet the requirements in routine care (e.g. insufficient insurance participation period). Such offers are referred to as innovative treatments in the following. Additional file 1 contains an exemplary overview of innovative treatments. The TIDieR checklist [19] in Additional file 2 provides a detailed description of the intervention.

**Table 1:** Stakeholders involved in the CC and the overall intervention process

Role	Role description
**Stakeholders involved in the Case Conference (CC)**	
Attending Primary Care Physician (PCP)	• Expert for his/her patients • Entered the patients on the digital communication platform and provided relevant medical information about them • Proposed and discussed the treatment option (decided in the CC) with the patient
Public Health Physician	• Employed by German pension insurance • Experienced in rehabilitation care planning • Could recommend appropriate treatment options from pension insurance services, such as rehabilitation or programmes for the participation in working life* like requalification or aids to support the performance of work (e.g., insoles)
Employee(s) from employment agencies or job centers	• Offered a non-clinical perspective • Could recommend appropriate support programmes from their portfolio, such as vocational training
**Other stakeholders involved in overall intervention process**	
Case administrator	• Employed by German pension insurance • Arranged the CC and the decided treatment programme for the patient (if agreed by the patient)
Social Worker	• Employed by German pension insurance • Could provide patients additional support (e.g. in accessing treatments)

*LTA (Acronym for ‘Leistungen zur Teilhabe am Arbeitsleben’)

Despite the early termination of the overall RehaPro-SERVE study, a process evaluation was conducted to explore the implementation of the intervention that had been completed, investigate the mechanisms of impact and to identify contextual factors [20]. Therefore, we aimed to answer the question of how the implementation of the digital case management approach for rehabilitation care planning is experienced by PCPs, patients, and other stakeholders involved in the intervention process, what contextual factors and mechanisms contribute to their perceptions and how these insights can inform future implementation strategies and approaches to rehabilitation care planning.

## Methods

This is a qualitative process evaluation using stakeholder interviews embedded within the RehaPro-SERVE study. Study procedures were tested in advance in a feasibility study [21]. Ethics approval was granted from the Faculty of Medicine Ethics Committee at the University Marburg (study reference 164/20). The reporting of this trial follows the consolidated criteria for reporting qualitative research checklist (COREQ [22], (Additional file 3).

### Sampling

Recruitment and data collection for the RCT began in September 2022 but were terminated early in December 2023 due to insufficient recruitment numbers. PCPs were enrolled in the study and tasked with recruiting six patients each, aged 40 to 60, with accumulated days of sick leave of at least four weeks in the last six months due to musculoskeletal, oncological or psychological conditions, or post-COVID-19 syndrome, and a high risk of early retirement (scores ≤ 36 on the Work Ability Index [17]). Exclusion criteria for patients included addiction or cranial brain trauma as a primary disease, an ongoing application for a rehabilitation treatment, being retired, receiving a retirement pension or disability benefits or ongoing application, being covered by private health insurance, working as a civil servant, permanently living abroad, the inability to speak and read sufficient German to read the study information and complete assessments and health issues that prevent participation in rehabilitation therapy. The occasion to talk about rehabilitative or other treatment options arose in the same way as usual, e.g. as a need identified by the PCP or upon a patient’s request. Eligible and consenting patients were randomised to the intervention or control group (treatment as usual). The recruitment rates for the RCT are also reported as part of the process evaluation.

All PCPs who had at least one patient recruited and randomised to intervention group, all intervention group patients as well as all other stakeholders from the CCs (a public health physician and employees from employment agencies or job centers), the case administrator and the social worker were invited to participate in the interview study. Potential interviewees were approached by telephone. Informed consent for the interview study was sought in writing and again verbally before and after the interview, to also ensure final agreement on the content of the interview.

### Data collection and management

Demographic characteristics of PCPs and patients were obtained as part of the RCT (Sept. 2022 to Dec. 2023). No demographic characteristics were collected for the stakeholders of the CCs.

Additional process data from the communication platform (duration of the CC in days, frequency and reasons for the activation of the social worker, decided treatment offers, decided innovative treatments) were extracted and analysed. Due to data protection regulations, the data in the communication platform was anonymous and could not be assigned to individual patients.

Semi-structured interviews were conducted between October 2023 and January 2024 (KB, VH). There were separate interview guidelines for PCPs, patients and the various roles in the CCs, containing similar but adapted questions. Interviews were arranged flexibly and held by telephone or occasionally one-to-one (location flexible according to the interviewee’s preferences, e.g., patients’ home). The interviews focused on the implementation and participants’ individual experiences with the intervention (the CCs) rather than on aspects of study conduct. Table 2 provides some examples of questions from the various interview guidelines. The complete interview guidelines (translated to English) are available in Additional file 4.

PCP interviews commonly ran for about 29 min (range 18–56 min), patient interviews for about 25 min (range 15–50 min) and the interviews with stakeholders involved averaged 40 min (range 23–54 min). Field notes provided context and relevant information outside of the interview. The interviews were recorded, transcribed with the help of automatic speech recognition [23], deidentified prior to analysis and loaded onto MAXQDA software [24] for data management. Upon request, one transcript was sent to the respective patient afterwards.

**Table 2:** Example questions from the various interview guidelines

Group	Example questions from the interview guideline
Primary care physicians	• How was your experience of the implementation of the case conference?
	• What helped/hindered the implementation?
	• What strengths/weaknesses do you see with regard to the case conference compared to standard care?
Patients	• How did you experience your (rehabilitation) application?
	• How was the cooperation with your primary care physician?
	• How did you feel about using the online platform?
	• If involved, how did you experience the support provided by the social worker?
Stakeholders involved in the process^1^	• In your opinion, are all important stakeholders involved?
	• How did you experience the cooperation between the stakeholders?
	• Which people do you think benefit most from the intervention?
	• What recommendations can be formulated for implementation based on your experiences?

^1^The stakeholders (case administrator, social worker, employment agency/job centre) also had their own, adapted interview guidelines. Some overlapping questions are listed here as examples

### Analysis

Demographic characteristics of the PCPs and patients who took part in the interview will be presented in tables. Descriptive analysis will be provided for process data from the communication platform.

Reflexive thematic analysis was used to identify, analyse and report the patterns (themes) in the data derived from the interviews [25, 26]. A staged process was performed including: familiarisation with data, generating initial codes, searching for themes, reviewing themes, defining and naming themes and producing the report [25]. After familiarisation with the data, three interviews per group (PCPs, patients and stakeholders from the CCs) were purposively selected in order to provide a broad spectrum. Initial coding was performed independently by KB and VH and at first separately for the three groups. The results and thoughts of this step were discussed and it was decided to analyse the groups together. A first coding guideline and initial themes were developed, tested again independently (KB, VH) on a further three interviews and refined afterwards. Final coding and development of themes was performed by KB alone in a reflexive process and in close dialogue with the research team. During the analysis it was decided to structure the emerging themes according to the Donabedian framework, stating that the quality of health care services can be defined by the interdependent components of structure, process and outcomes [27]. Accordingly, structure can be defined as the setting in which health care takes place (e.g., resources or organisational structures) or as ‘having the right things’, processes as all actions while providing and receiving healthcare (e.g., interpersonal actions) or as ‘doing things right’ and outcomes as the results of health care (e.g., health status or satisfaction) or as ‘having the right things happen’ [27, 28]. We considered this model to be well aligned with the principle questions for conducting a process evaluation: what was implemented and how, how does the implemented intervention lead to change, and how do contextual factors influence implementation and outcomes [20], while it has also shown to be applicable in the evaluation of eHealth interventions [29].

When coding, only aspects relating to the intervention (i.e. the application process and the treatment offer) were included. Aspects relating to the approval process and the treatment itself were deliberately excluded from the analysis, as they lie outside the scope of the intervention.

### Research team conducting interviews and analysing data

The interviews were conducted by KB and VH and analysed by KB, VH and VvdW (all female). KB and VvdW are both psychologists, VvdW with a PhD, and employed as research fellows. While KB has limited experience, VvdW is experienced in qualitative research methods. VH is a research assistant with a degree in health sciences. Both interviewers, in some cases, already interacted with participating PCPs and/or patients in the context of recruitment or data collection. One participating PCP was already known to the interviewers in the form of a work relationship.

## Results

### Sample

From 525 approached PCP practices a total of 24 PCPs (4.6%) could be recruited for the RCT. 33 patients consented in writing to being contacted by the study team (other interested patients called the study team themselves, but their number could not be adequately documented), from which 20 (60.6%) were recruited successfully (ten patients were not eligible for inclusion, two patients could not be contacted and one patient declined to participate due to a recent severe diagnosis). 10 patients each were assigned to the two groups (intervention and control group).

We planned on approaching all PCPs who had at least one patient recruited and randomised to intervention group, all intervention group patients as well as all other stakeholders involved in the process to participate in the interview study. One of the ten intervention group patients was excluded from the RCT before the start of the CC due to not meeting the inclusion criteria, and was therefore not asked to participate in the interview as the person had not progressed through the CC (as well as the attending PCP). Due to staff turnover, the study’s public health physician was no longer available and therefore also not asked to participate in the interview.

Eight PCPs, nine patients and five stakeholders involved in the CC or the overall process were approached. One PCP declined to participate for unknown reasons. One PCP and two patients were no longer contactable. A total of *n* = 18 interviews were conducted. The sample consisted of six PCPs (Table 3), seven patients (Table 4) and five stakeholders involved: the case administrator, the social worker and three employees from the employment agencies or job centers. In one patient interview, the spouse also participated in the interview due to the patient’s poor state of health.

**Table 3:** Characteristics of participating PCPs

PCP	Sex	Age (years)	Work experience in primary care (years)	Working hours	Practice type	Patients under care
1	F	62	33	Full-time	Individual practice	Patient 1
2	M	65	30	Full-time	Joint practice	-
3	F	34	5	Full-time	Joint practice	Patient 3
4	M	56	23	Full-time	Supraregional professional practice association (ÜBAG)	Patient 4
5	F	32	< 1	20 h/w	Supraregional professional practice association (ÜBAG)	Patient 5
6	M	39	9	Full-time	Joint practice	Patient 6, Patient 7

**Table 4:** Characteristics of participating patients

Patient	Sex	Age (years)	Social situation	Main diagnosis^1^	WAI^2^	Attending PCP
1	M	49	three-person household (no children)	musculoskeletal	35	PCP 1
2	F	48	two-person household (incl. one child)	musculoskeletal	8	-
3	M	47	three-person household (incl. one child)	musculoskeletal, psychological	20	PCP 3
4	F	50	three-person household (incl. one child)	psychological, post-COVID-19	8	PCP 4
5	F	51	two-person household (no children)	post-COVID-19	11	PCP 5
6	M	48	four-person household (incl. two children)	musculoskeletal	26	PCP 6
7	F	57	Living alone	musculoskeletal, psychological	8	PCP 6

^1^single choice item, some patients however gave multiple answers

^2^WAI Work Ability Index (Scores from 7 to 49), only patients included with scores ≤ 36

### Process data from the communication platform

The average duration of the CC process was 14 days (range 9–22 days). The ‘activation’ of the social worker in the CCs was not carried out reliably. Although a need for additional patient support was identified in the CC, the relevant button on the platform was often not clicked (e.g., because it was overseen or forgotten to be done). As a workaround, the social worker was involved in the process without official ‘activation’ if necessary and ended up to be active in all cases to some extent. Reasons for ‘activation’ were to secure the project processes, support in filling out questionnaires, guidance on the course of the study and giving advice/counselling on possible treatments and the selection of an institution (rehabilitation clinic). All of the treatment programs decided upon included inpatient rehabilitation with a different focus, depending on the specific condition. Only one innovative treatment was agreed, which included app-based aftercare as a supplement to inpatient rehabilitation. Moreover, one PCP stated not having participated in the CC herself due to problems with the technology, despite receiving training from the study team and providing a treatment proposal.

### Qualitative results

Eight themes with subthemes emerged: overall concept, stakeholder involvement, technical realisation/platform, interpersonal interactions, (un-)clarity of the process, time aspects, patients` satisfaction and benefit. Based on the Donabedian framework [27] these themes were grouped under three main categories and will be presented accordingly: *intervention structure*,* intervention process and intervention outcomes* (Table 5).

**Table 5:** Themes and subthemes

Themes	Subthemes
***Intervention structure***	
1. Overall concept	
2. Stakeholder Involvement	• Involvement of all relevant stakeholders in the CC • Involvement of social worker
3. Technical realisation / platform	
***Intervention process***	
4. Interpersonal interactions	• Collaboration • Decision-making process in the CC • Support from social worker
5. Clarity of the process	
6. Time aspects	• Personal effort and time required • Duration of the application process
***Intervention outcomes***	
7. Patients’ satisfaction	• With the application process • With treatment offer
8. Benefit	

### Intervention structure

#### Overall concept

The overall concept and the idea of bringing together different stakeholders in the CCs were considered positive in general.

*... I also thought the concept you came up with was quite good. (Patient 7)*

*Because basically, I think it’s a great idea... (PCP 4)*

*I think the case conference as such, with this idea of bringing different stakeholders together, is so good that it should definitely be continued in some way. (Employment agency/Job center 1)*

PCPs particularly appreciated the opportunity to offer patients access to innovative treatments. However, some PCPs did not share this opinion and did not perceive any need to adapt the application process. With regard to a potential continuation, there was further a wish for a case manager to support the entire application process.

... *so a middleman who is really in dialogue with everyone*,* with all the institutions involved*,* and can also give feedback on whether things are going well or not … (PCP 3)*.

#### Stakeholder involvement

##### Involvement of all relevant stakeholders in the CC

There were different opinions about whether or not all relevant stakeholders were involved in the CC. Persons interviewed from all groups expressed the wish for greater involvement of the patients themselves to have the opportunity to express their wishes. The social worker also wished for more involvement and suggested the idea of serving as an intermediary for the patient.

*Well*,* I thought there were a lot of people involved*,* but I found that the patient herself wasn’t involved that much. (PCP 5)*

PCPs and stakeholders in the CCs also wished for direct communication and contact with treatment funders and the persons responsible for approval in order to increase transparency.

*So*,* there is no one at the table who actually authorises or rejects a treatment afterwards. I think that’s a pity. (Employment agency/Job center 1)*

##### Involvement of social worker

The general involvement of the social worker was viewed as thoroughly positive. The social worker herself considered her role to be essential for the application process as part of the study, estimating a high need for support and indicating she was involved in almost all cases. In general, however, she expressed that she did not have access to enough information about the patients to prepare before contacting patients. Patients were happy to have human support for the application:

*Is there also someone on hand for a normal rehabilitation application? Like a social worker? No*,* probably not. You see*,* that’s the difference. (Patient 5)*

#### Technical realisation/platform

Some technical problems with the platform, such as instability and display errors, were reported during the course of the study. Overall, however, these had decreased over time.

*These are mostly technical problems that people don’t … have problems logging in somehow or that they can’t see something*,* that some documents can’t be uploaded*,* these are the classic problems that have arisen. (Case administrator)*

Opinions were divided on the user-friendliness of the platform. While some had no problems with usability, others described the platform as unintuitive and confusing. In some cases, it was noted that a regular use of the platform would make it easier to use.

*Maybe I didn’t familiarise myself with it enough*,* but until the end I had the feeling that I didn’t really understand (the platform) completely. It wasn’t very intuitive*,* these different levels*,* the clicking*,* case conference*,* suddenly it started running. (PCP 6)*

Overall, the training provided by the study team to PCPs and the support given to patients by the social worker were important for using the platform and ensuring that the processes ran properly. Moreover, the communication between the stakeholders via the platform was experienced as limited. Among other things, a chat function and the possibility of meeting online in the form of a conference were suggested as future developments for the platform. Overall, it became clear that some form of digital solution is desired, but that there is a need for adjustments to the current form. Moreover, one PCP reported that she did not take part in the CC herself due to problems with the technology after receiving training from the study team and providing a treatment proposal.

### Intervention process

#### Interpersonal interactions

##### Collaboration

The collaboration between the stakeholders in the CC was perceived overall as positive and solution-orientated. PCPs also found the exchange in the CC to be generally interesting. However, despite training with the study team, in some cases the PCPs were not aware of any form of collaboration on the platform. In general, the opportunities for exchange within the platform were perceived as too limited. There was a desire for more opportunities for exchange, such as the option of video conferencing, especially for complex cases.

*I thought it was good to read the different things that people wrote … to hear different opinions again*,* but I would have liked to see more dialogue*,* which is what I had initially imagined. That there would be more exchange than*,* two messages and that’s it. (PCP 5)*

Collaboration between doctors and patients was also generally perceived as positive, as well as the cooperation with the study team.

##### Decision-making process in the CC

Some PCPs were critical of the collaborative decision-making process. For example, one PCP reported not having taken part in the CC herself, while others felt that the scope of the decision-making process was too limited. Employees from employment agencies or job centers, on the other hand, reported predominantly positively and of a quick and consensual decision-making process. Patients generally perceived that their wishes and needs were taken into account.

*So*,* what I had read from the other stakeholders was finished*,* complete suggestions and I didn’t have the feeling that it was a completely joint approach*,* but rather that one person said something and then we said it was okay. (PCP 5)*

##### Support from social worker

The support given by the social worker was both organisational and emotional and was perceived as particularly valuable by patients. The support was organised flexibly and resembled case management (mediating position, ensuring the process and information patients about progress), which was generally wished for (see above).

*I felt so well cared for. (Patient 2)*

#### (Un-)Clarity of the process

Overall, several problems arose regarding the clarity of the process of the intervention. PCPs were partly unclear about which other stakeholders were involved in the CC.

*I honestly have to say that I didn’t realise that anyone other than me was involved. I filled it in. I also saw that it was written something. But*,* as I said*,* I didn’t realise that anyone else was involved apart from me. (PCP 3)*

In addition, some PCPs were not aware of the option of deciding on innovative treatments rather than traditional rehabilitation therapies although this was explained in the training provided by the study team. Additional file 1 contains an exemplary overview of such innovative treatments. Many patients were also unaware of this information. The PCPs perceived the entire process, also with regard to the technical implementation, as sometimes too complicated and confusing. Patients reported in some cases that they were unaware that a CC was taking place or lacked information about what was discussed and decided at the conference. The social worker had a central part in providing patients further education and information about the process (see above). For PCPs this part was done by the study team if questions arose.

*... she (social worker) always explained and told me all the processes and I always thought that was really good. (Patient 7)*

#### Time aspects

##### Personal effort and time required

Questionnaires that had to be completed in the platform for decision-making in the CC were sometimes considered too extensive by patients. PCPs reported an overall increase in workload due to the changed application process. Only a few cases reported a facilitation through prepared and pre-filled applications.

*It was a bit more work for me initially. Erm*,* but I hope that the patient will now be helped more individually. (PCP 4)*

##### Duration of the application process

Patients predominantly reported being content with the overall duration of the application process. However, the social worker stated that many proceedings had delayed a quick process.

*So that (the application process) went really quickly and that (...) was positive. (Patient 1)*

### Intervention outcomes

#### Patients`satisfaction

##### Patients’ satisfaction with the application process.

All patients were very satisfied with their application process.

*I was satisfied … the application process went quickly; someone came to support me and then it all went out. Everything went well with the application. (Patient 3)*

##### Patients’ satisfaction with treatment offer.

Patients were also satisfied with the treatment offered, and were able and willing to participate in it as proposed.

#### Benefit

Opinions were divided as to whether and, if so, what advantage the intervention offers in comparison to standard care. Several interviewees were positive about the fact that patients benefited from being guided and supported during the intervention. At the same time, there was hope that the different perspectives on the patient would lead to a more tailored treatment planning. Some PCPs reported that in their cases they had seen no benefit (neither for them nor for the patients) while their workload had increased. Overall, it became clear that an exchange between the various stakeholders and the CC is considered particularly useful in more complex cases.

*I can definitely see the potential for it to be a better way of submitting an application than what I’ve normally done as a rehabilitation application so far. But I don’t think it has to be necessary for every patient. (PCP 5)*

## Discussion

An interdisciplinary case management intervention for rehabilitation care planning was developed to facilitate the access to medical rehabilitation. To understand the implementation of the intervention and to identify contextual factors as well as mechanisms for a successful implementation in the context of primary care a qualitative process evaluation was conducted.

While the idea of a digital case management procedure involving PCPs, relevant staff from the employment agency/job centre and a public health physician was considered beneficial by stakeholders as well as patients, the intervention could not be implemented successfully across the board. Several factors were responsible: first, patients and the social worker who had direct contact with the patients were not part of the decision-making process regarding the treatment, which made it more difficult to take patients’ preferences into consideration. Second, the digital platform had technical issues and was not always experienced as user-friendly. Third, for PCPs, the collaboration with the other stakeholders on the platform was perceived as limited, some of them were not aware of the option to discuss treatment options via the platform. They also perceived their influence on treatment decisions as limited. On the other hand, employment agency/ job centre stakeholders engaged in the decision process and experienced the use of the platform as positive. Lastly, PCPs perceived intervention procedures as unclear and time consuming. However, all patients approved of the application process, in particular when supported by the social worker. This additional support and the presence of a person they had personal contact with was particularly valued by the patients. The involvement of different stakeholders in form of a CC was seen as especially useful for more complex cases.

Patients are not involved in the current paper-based application procedure for rehabilitation treatment, which was transformed to a digital format and complemented by innovative treatment options for this project. The results of our study showed that the lack of patient participation was universally regarded as a shortcoming. Shared decision-making (SDM) is the collaborative decision making process involving the clarification that a decision is needed, a discussion of options and exploration of patient preferences as well as the treatment decision itself, is nowadays largely seen as an integral part of health care [30]. Given the discussion of different treatment options in the digital CCs and the impact of patient preferences on the treatment decisions, a SDM approach would be appropriate for this type of decision [31].

The Donabedian framework [27] provided a suitable basis for structuring the qualitative results. With the consideration that patients should participate more in the intervention, the model could also be broadened to a person-centred approach [32].

The technical issues stakeholders experienced with the platform were partly due to the software itself, particularly at the start of the study. Some issues, such as interface problems between the platform and the software used in primary care practices (e.g. to upload patient related information (e.g. test results)) remained throughout the study. Training was provided by the study team but PCPs used the platform only a few times. Repeated use and resulting familiarisation could help stakeholders to feel more comfortable and competent using the platform. Overall, it became clear that a digital transformation of the application process is generally favoured, but that adjustments to the current form are necessary.

While the involvement of PCPs in the interprofessional collaboration was enabled by the platform and the steps to do so were part of the training, PCPs were not always aware of this option. Additional training, such as the opportunity to complete the online processes with example cases, might facilitate the use of the platform. Although PCPs received support to enter the data for each patient up to the point that they offered their treatment suggestion, they had to continue the discussion of the treatment at a later point in time by themselves. This was not always evident to PCPs. Therefore, future versions of the platform should aim to optimise user-friendliness and visibility of features to ensure minimal time investment from stakeholders. In particular, as lack of time has been shown to be a barrier to interprofessional collaboration in primary care practices [33]. Sending automated push notifications though the platform should also be considered as a tool to make it more efficient when further input is required or an option.

### Strengths and limitations

The study investigated a novel digital platform for rehabilitation applications that combined interprofessional CCs with the digitalisation of the application process and a wider range of innovative and individualised treatment options including work-related support (see Additional file 1). From the patients’ perspective, the intervention was a positive experience. However, the study had several limitations: First, initially, recruitment was severely disrupted due to the Covid-19 pandemic and even afterwards not progressing as planned. The small sample size of patients and PCPs might have limited the range of views expressed in this interview study. The small sample size of patients also led to a limited experience with the digital platform by the PCPs, more patients would have provided more engagement in the CC and therefore more familiarity with the system. Second, we could not interview the public health physician working on the project as he had moved to a different post and could not be reached. Therefore, his views are not presented in this study. Given the social worker and case administrator were part of the larger research group, they were known to the interviewers, in addition, the PCPs and/or patients were already in contact with the interviewers as part of the recruitment and data collection process. Last, the process evaluation was completed by the same team that was responsible for the RCT. While this provided a comprehensive knowledge of the intervention and the trial, it might have limited the objectivity when completing the interviews and analysis.

## Conclusion

A digitalisation of the application procedure for rehabilitation and further treatment options is acceptable to patients with psychological, oncological and musculoskeletal health issues or post-COVID-19 syndrome especially if the support of a social worker is available. The digital platform to share data and discuss treatment options requires sufficient training and needs to be user-friendly to ensure the application process is timesaving and not time-consuming for PCPs. In particular, the process of discussion of treatment options needs to be visible and easy to access for the PCPs. Patients should be included in the CC to discuss their treatment options, as an early consideration of their preferences would increase efficiency of the process.

## Electronic supplementary material

Below is the link to the electronic supplementary material.

Supplementary Material 1.

Supplementary Material 2.

Supplementary Material 3.

Supplementary Material 4.

## Data Availability

The deidentified transcripts (in German) are available from the corresponding author on reasonable request.

## Abbreviations

CC: Case conference
PCP: Primary care physician
SDM: Shared-decision making
WAI: Work ability index
