# Supplementary material for: Lessons learned from implementing a digital rehabilitation care planning platform to improve care access for patients with work disability: qualitative process evaluation of the RehaPro-SERVE study
Source: BMC Health Serv Res. 2024 Oct 29;24:1299. doi: 10.1186/s12913-024-11778-3 (PMC11520423; doi:10.1186/s12913-024-11778-3)
Supplement: Supplementary file 1 — Supplementary Material 1. [file 12913_2024_11778_MOESM1_ESM.docx]

**Additional File 1: Exemplary overview of innovative forms of care** **‘innovative treatments****’**

**Article**: Lessons learned from implementing a digital rehabilitation care planning platform to improve care access for patients with work disability: Qualitative process evaluation of the RehaPro-SERVE study

**Authors**: Kristina Buch, Viktoria Hamme, Annette Becker, Ulf Seifart, Catharina Maulbecker-Armstrong, Karin Moser, Pellumbesha Seferi, Antonia Keller, Veronika van der Wardt

1. **Rehabilitation before surgery**

E.g. Rehabilitation before operation.

1. **Sequential rehabilitation**

E.g. Implementation of sequential therapy in e.g. 3 blocks of 1 week each, over a period of 6-9 months instead of a three-week therapy.

1. **Innovative aftercare services**

E.g. Sports programmes near to the patient's home after rehabilitation

1. **Caspar Health**

A digital rehabilitation service, which could be recommended as an innovation without prior treatment or rehabilitation

1. **Implementation of already established rehabilitation programmes or programmes for the participation in working life** (LTA ‘Leistungen zur Teilhabe am Arbeitsleben’) **at different time points**
2. **Already establishes rehabilitation or programmes for the participation in working life** (LTA ‘Leistungen zur Teilhabe am Arbeitsleben’) **for which patients would not meet the requirements in routine care** (e.g. due to insufficient insurance participation period)
3. **Implementation of support programmes run by employment agencies or job centres**

E.g. Vocational training
