# Supplementary material for: Lessons learned from implementing a digital rehabilitation care planning platform to improve care access for patients with work disability: qualitative process evaluation of the RehaPro-SERVE study
Source: BMC Health Serv Res. 2024 Oct 29;24:1299. doi: 10.1186/s12913-024-11778-3 (PMC11520423; doi:10.1186/s12913-024-11778-3)
Supplement: Supplementary file 2 — Supplementary Material 2. [file 12913_2024_11778_MOESM2_ESM.docx]

**Additional File 2:**

**Intervention description based on TIDieR checklist^[[1]](#footnote-1)^**

**Article**: Lessons learned from implementing a digital rehabilitation care planning platform to improve care access for patients with work disability: Qualitative process evaluation of the RehaPro-SERVE study

**Authors**: Kristina Buch, Viktoria Hamme, Annette Becker, Ulf Seifart, Catharina Maulbecker-Armstrong, Karin Moser, Pellumbesha Seferi, Antonia Keller, Veronika van der Wardt

| Name | RehaPro-SERVE – digital case management platform |
| --- | --- |
| Why? (rationale) | The digital case management approach aims to facilitate rehabilitation care planning. The quality of care and access to rehabilitative or other services to promote return to work is supposed to be improved by various components:  1) efficient interdisciplinary communication and joint decision making among stakeholders (attending primary care physician, a public health physician and an employee of the employment agency or job centre) in a case conference (CC). The stakeholders propose various treatment options, which are then discussed until a consensus on a suitable treatment programme is reached. The CCs as well as agreed treatments are arranged by a case administrator. The digital platform enables fast, time-flexible, written communication without the need for a meeting.  2) tailoring of treatment offers. Care planning is individualised, tailored to the patient's needs and based on flexible clinical decisions. The intervention enables the provision of regular or innovative treatments. Innovative treatments can consist of services from the employment agencies or job centres (e.g. occupational therapy, return-to-work support or work-related educational courses), that are usually not funded by the German pension insurance. Patients can also receive treatments, for which they do not meet the requirements in routine care (e.g. insufficient insurance participation period). Treatments may be arranged on an in- and/or out-patient basis.  3) possibility for additional patient support by a social worker, decided by the primary care physician (PCP) or in the CC based on the patient's needs. |
| What? (procedure) | The PCP enters the patient’s relevant medical information on the digital communication platform and make an initial proposal for an appropriate treatment programme. All stakeholders involved in the CC are notified, are able to access the information and to propose alternative treatment options. Afterwards treatment options are discussed in writing, until a consensus is reached on a suitable programme. The PCP proposes the decided treatment programme to the patient. If the patient consents, the treatment programme will be arranged by the case administrator. A social worker can be involved to support the patient. Support will be aligned with the needs of the patient and may include assistance with application procedures, such as filling out questionnaires, helping the patient with coordinating treatments, appointments or transport or planning absences from the family. |
| Who provided? | - PCPs are experts for their patients, enter the patient’s relevant medical information on the digital communication platform and make an initial proposal for an appropriate treatment programme. Once the discussion reaches consensus, the PCP discusses the decided treatment programme with the patient. - A case administrator, employed by the German pension insurance to facilitate stakeholder communication and support technical processes if necessary. If the patient consents to the treatment proposal from the CC, the case administrator arranges the programme. - A public health physician, also employed by the German pension insurance and experienced in rehabilitation care planning, attends the CCs and can recommend treatments from pension insurance services. - (At least one) employee of the employment agency or job centre also participates in the CC and can offer services from the employment agencies or job centres (e.g. occupational therapy, return-to-work support or work-related educational courses), which are usually not funded by the German pension insurance. - A social worker can be involved to offer additional support to patients. The social worker is also employed by the German pension insurance and is not involved in the CC itself. |
| How? | Communication on the digital platform is in writing. The PCP enters the patient’s relevant medical information on the digital communication platform, which can be accessed electronically by the stakeholders involved in the CC. All stakeholders can propose treatments, which are discussed afterwards until a consensus on a suitable treatment programme is reached. |
| Where? | CCs takes place on the digital communication platform. |
| When and how much? | The CC begins with the PCP entering the patient’s relevant medical information on the platform end as soon as the discussion reaches a consensus. The CC can be reconvened until the treatment is considered completed by the stakeholders. The frequency of communication and CCs will be flexible and individually tailored to the patient’s case. |
| Tailoring | Treatment programmes are tailored to the patient's needs and based on flexible clinical decisions. |
| How well? (adherence/fidelity) | Within the study n=9 CCs took place. The average duration of CCs was 14 days (range 9-22 days). The activation of the social worker was not carried out reliably. The social worker was active in all cases to some extent. Reasons for activation were to secure the project processes, support in filling out questionnaires, guidance on the course of the study and giving advice/counselling on possible treatments and the selection of an institution (rehabilitation clinic). All of the treatment programmes decided upon included inpatient rehabilitation with a different focus, depending on the specific condition. Only one innovative treatment was agreed, which includes app-based aftercare as a supplement to inpatient rehabilitation. One PCP reported that she did not take part in the CC herself due to problems with the technology after receiving training from the study team and providing a treatment proposal. |

1. Hoffmann T C, Glasziou P P, Boutron I, Milne R, Perera R, Moher D et al. Better reporting of interventions: template for intervention description and replication (TIDieR) checklist and guide BMJ 2014; 348 :g1687 doi:10.1136/bmj.g1687 [↑](#footnote-ref-1)
