# Supplementary material for: Lessons learned from implementing a digital rehabilitation care planning platform to improve care access for patients with work disability: qualitative process evaluation of the RehaPro-SERVE study
Source: BMC Health Serv Res. 2024 Oct 29;24:1299. doi: 10.1186/s12913-024-11778-3 (PMC11520423; doi:10.1186/s12913-024-11778-3)
Supplement: Supplementary file 4 — Supplementary Material 4. [file 12913_2024_11778_MOESM4_ESM.docx]

**Interview guideline for patients**

**Introduction**

*Thank you for agreeing to do this interview with me. We are interested in how you experienced participating in the study, particularly* ***the application process****. This is about your personal opinion and there are no "right" or "wrong" answers. The interview will last approximately 30-60 minutes. However, you can interrupt the interview at any time for a break or end it completely. I have brought enough time with me and will adapt to you.*

*I will record our conversation with this digital device so that we can analyse the results scientifically afterwards. The recording will be transcribed and pseudonymised so that it cannot be directly attributed to your name or personal data. Do you agree to this? I will ask you again after the interview has been completed whether you still agree to this. Do you have any questions? Then let's start now.*

**If you think about it (independently of your participation in the study), what would you wish for an ideal application process for rehabilitation?**

**How did you (now) experience your (rehabilitation) application?**

- How was decided which (rehabilitation) treatment(s) you would receive?
- How did you find out which (rehabilitation) treatment(s) you should receive?
- Did you feel involved in the treatment planning?
- How was the cooperation with your primary care physician?
- What did you like about the overall process?
- What didn't you like about the overall process?
- Were there any difficulties or problems?
- What would you have wished differently? / What could be improved in the processes?
- How did you feel about using the online platform?
  - How did you manage to log in to the platform?
  - How did you experience completing the online questionnaires (only the questionnaires on the platform)?
- How did you experience the duration of the (rehabilitation) application process?
  - How do you rate it in comparison to a regular rehabilitation application?
  - To what extent do you think this has affected your ability to work?
- How satisfied are you overall with your (rehabilitation) application process?
  - If you had to express this in figures, how would you rate your satisfaction with the application process on a scale of 1-10 (1=not at all satisfied; 10=completely satisfied)?

**Now I would like to know about the outcome of the case conference, i.e. the treatment proposal** (if I have understood correctly, this was XY for you).

- To what extent did you experience the outcome of the case conference (i.e. the discussion between your family doctor, the pension insurance company and the employment agency/job centre) as having an open/flexible outcome?
  - To what extent were you aware that other treatments (apart from rehabilitation) could also be decided in the case conference?
- Were you able/willing to accept the treatment offer as proposed?
- Was the social worker involved in your process?
  - How did you experience the support provided by the social worker?
- Do you believe that your (rehabilitation) programme(s) have had an effect on your work incapacity? In what way? / Why not?

**Conclusion**

*Finally, do you have any suggestions / criticisms / other issues you would like to raise? Do you still agree that this recording may be used for our scientific evaluation?*

**Thank you very much for the interview!**

**Interview guideline for primary care physicians**

**Introduction**

*Thank you for agreeing to do this interview with me. We are interested in how you evaluate the work with the case conference and its benefits. This is about your personal opinion and there are no "right" or "wrong" answers. The interview will last approximately 30-60 minutes. However, you can interrupt the interview at any time for a break or end it completely. I have brought enough time with me and will adapt to you.*

*I will record our conversation with this digital device so that we can analyse the results scientifically afterwards. The recording will be transcribed and pseudonymised so that it cannot be directly attributed to your name or personal data. Do you agree to this? I will ask you again after the interview has been completed whether you still agree to this. Do you have any questions? Then let's start now.*

**Initial situation**

- What would you wish for an ideal application process for rehabilitation (independent of the study)?
- Why did you take part in the study? *(Is the problem being recognised and experienced as significant?)*
- What expectations did you have of participating in the study or the intervention?

**Realisation**

- How was your experience of the implementation of the intervention?
  - Were there any difficulties or problems?
- What helped the implementation?
- What hindered the implementation?
- In your opinion, are all important stakeholders involved in the implementation?
  - Who is missing? Why?
- How did you experience the cooperation of the stakeholders (in the case conference/intervention)?
  - How did you experience the joint decision-making process?
- How did you proceed with the initial treatment proposal?
- In which cases was the social worker activated?
  - For what reasons?
  - How did you experience the support provided by the social worker?
- Were innovative or flexibilised services decided in the SMK?
  - Can you tell me more about these?
- How did you experience using the online platform?
  - Were there any difficulties or problems?
  - How well did the process of entering patients to platform work?

**Evaluation**

- Has anything changed as a result of the case conference/intervention? How would you rate the success/failure?
  - To what exactly do you attribute the success/failure?
  - What influence does the joint development of a treatment concept in the case conference have?
  - What influence does the innovative/flexible treatment concept have?
- What strengths/weaknesses (*advantages/disadvantages*) do you see with regard to the case conference/intervention compared to standard care?
- Which people do you think benefit most from the intervention?
- How successful was the implementation of the intervention/case conference in terms of time (outside of participation in the study)? Is there a time saving?
- Were your expectations of the study/intervention fulfilled?
  - In what way?
  - Why not?
- What feedback do you receive from patients?
- How would you rate the satisfaction of the patients (intervention group) with the treatment programme?

**Outlook**

- What suggestions/wishes arise from your experiences for a possible continuation?
- What recommendations can be formulated for implementation based on your experiences?
- What needs to change?

**Conclusion**

*Finally, do you have any suggestions / points of criticism / further topics that you would like to address? Do you still agree that this recording may be used for our scientific evaluation?*

**Thank you very much for the interview!**

**Interview guideline for the case administrator**

**Introduction**

*Thank you for agreeing to do this interview with me. We are interested in how you evaluate the case conference and its benefits. This is about your personal opinion and there are no "right" or "wrong" answers. The interview will last approximately 30-60 minutes. However, you can interrupt the interview at any time for a break or end it completely. I have brought enough time with me and will adapt to you.*

*I will record our conversation with this digital device so that we can analyse the results scientifically afterwards. The recording will be transcribed and pseudonymised so that it cannot be directly attributed to your name or personal data. Do you agree to this? I will ask you again after the interview has been completed whether you still agree to this. Do you have any questions? Then let's start now.*

- Can you explain your role in the study?

**Realisation**

- How did you experience the implementation of the intervention?
  - Were there any difficulties or problems?
- What helped the implementation?
- What hindered the implementation?
- In your opinion, are all important stakeholders involved in the implementation?
  - Who is missing? Why?
- How did you experience the cooperation between the stakeholders (in the case conference)?
  - To what extent has cooperation changed over time?
- How did you experience the use of the online platform?
  - Were there any difficulties or problems?

**Evaluation**

- How did you experience the implementation of the intervention/case conference in terms of time?

**Outlook**

- What suggestions/wishes arise from your experiences for a possible continuation?
- What recommendations can be formulated for implementation based on your experiences?
- What needs to change?

**Conclusion**

*Finally, do you have any suggestions / points of criticism / further topics that you would like to address? Do you still agree that this recording may be used for our scientific evaluation*?

**Thank you very much for the interview!**

**Interview guideline for the social worker**

**Introduction**

*Thank you for agreeing to do this interview with me. We are interested in how you evaluate the case conference and its benefits. This is about your personal opinion and there are no "right" or "wrong" answers. The interview will last approximately 30-60 minutes. However, you can interrupt the interview at any time for a break or end it completely. I have brought enough time with me and will adapt to you.*

*I will record our conversation with this digital device so that we can analyse the results scientifically afterwards. The recording will be transcribed and pseudonymised so that it cannot be directly attributed to your name or personal data. Do you agree to this? I will ask you again after the interview has been completed whether you still agree to this. Do you have any questions? Then let's start now.*

- Can you explain your role in the study?

**Realisation**

- In which cases have you been activated as a social worker?
  - For what reasons?
  - How were you activated?
- What did the support for patients look like exactly?
  - What issues did you support them with?
  - How would you describe or rate the importance your role as a social worker?
- In your opinion, were all the important stakeholders involved in the implementation?
  - Who is missing? Why?
- How did you experience the cooperation with the other stakeholders (e.g. case administrator, PCPs)?

**Evaluation**

- Did the intervention change anything for the patients? How would you rate the success/failure?
- To what exactly do you attribute the success/failure?
- What strengths/weaknesses (*advantages/disadvantages*) do you see with regard to the intervention compared to standard care?
- Which people do you think benefit most from the intervention?
- What feedback do you receive from patients?
- How would you rate the satisfaction of the patients (intervention group) with the treatment programme?

**Outlook**

- What suggestions/wishes arise from your experiences for a possible continuation?
- What recommendations can be formulated for implementation based on your experiences?
- What needs to change?

**Conclusion**

*Finally, do you have any suggestions / points of criticism / further topics that you would like to address? Do you still agree that this recording may be used for our scientific evaluation*?

**Thank you very much for the interview!**

**Interview guideline for employees of the employment agency/job center**

**Introduction**

*Thank you for agreeing to do this interview with me. We are interested in how you evaluate the work with the case conference and its benefits. This is about your personal opinion and there are no "right" or "wrong" answers. The interview will last approximately 30-60 minutes. However, you can interrupt the interview at any time for a break or end it completely. I have brought enough time with me and will adapt to you.*

*I will record our conversation with this digital device so that we can analyse the results scientifically afterwards. The recording will be transcribed and pseudonymised so that it cannot be directly attributed to your name or personal data. Do you agree to this? I will ask you again after the interview has been completed whether you still agree to this. Do you have any questions? Then let's start now.*

- Can you explain your role in the study?
- What were your expectations towards the case conference?

**Realisation**

- How did you experience the case conference?
  - Were there any difficulties or problems?
- What helped the implementation?
- What hindered the implementation?
- In your opinion, are all important stakeholders involved in the implementation?
  - Who is missing? Why?
- How did you experience the cooperation of the stakeholders in the case conference?
  - How did you experience the joint decision-making process?
- How did you proceed when submitting a treatment proposal?
- To what extent did you have the impression that your suggestions were incorporated into the treatment programme?
- Were innovative or flexibilised services decided in the case conference?
  - Can you tell me more about these?
- How did you experience using the digital platform?
  - Were there any difficulties or problems?

**Evaluation**

- How would you rate the success/failure of the case conference?
  - To what exactly do you attribute the success/failure?
  - What influence does the joint development of a treatment concept in the case conference have?
  - What influence does the innovative/flexible treatment concept have?
- What strengths/weaknesses (*advantages/disadvantages*) do you see with regard to the case conference compared to standard care?
- Which people do you think benefit most from the case conference?
- How did you manage to integrate the case conference into your daily work routine?
- Were your expectations of the case conference fulfilled?
  - In what way?
  - Why not?

**Outlook**

- What suggestions/wishes arise from your experiences for a possible continuation?
- What recommendations can be formulated for implementation based on your experiences?
- What needs to change?

**Conclusion**

*Finally, do you have any suggestions / points of criticism / further topics that you would like to address? Do you still agree that this recording may be used for our scientific evaluation*?

**Thank you very much for the interview!**
